# Comparative analysis of C-heterochromatin, ribosomal and telomeric DNA markers in chromosomes of Pamphagidae grasshoppers from Morocco

**DOI:** 10.3897/CompCytogen.v13i1.32039

**Published:** 2019-02-27

**Authors:** Olesya G. Buleu, Ilyas Y. Jetybayev, Dragan P. Chobanov, Alexander G. Bugrov

**Affiliations:** 1 Novosibirsk State University, Pirogova Str. 2, Novosibirsk 630090, Russia Institute of Systematics and Ecology of Animals, Russian Academy of Sciences Novosibirsk Russia; 2 Institute of Systematics and Ecology of Animals, Russian Academy of Sciences, Siberian Branch, Frunze str. 11, 630091 Novosibirsk, Russia Novosibirsk State University Novosibirsk Russia; 3 Institute of Cytology and Genetics, Russian Academy of Sciences, Siberian Branch, Pr. Lavrentjeva 10, 630090 Novosibirsk, Russia Institute of Cytology and Genetics, Russian Academy of Sciences Novosibirsk Russia; 4 Institute of Biodiversity and Ecosystem Research, Bulgarian Academy of Sciences, Tsar Osvobodotel Boul. 1, Sofia 1000, Bulgaria Institute of Biodiversity and Ecosystem Research, Bulgarian Academy of Sciences Sofia Bulgaria

**Keywords:** Pamphagidae grasshoppers, karyotypes, C-banding, FISH, telomeric repeats (TTAGG)_n_, ribosomal DNA repeats

## Abstract

The karyotypes and the localization of C-bands, clusters of ribosomal DNA and telomeric repeats of 10 species of the family Pamphagidae from Morocco are described for the first time. The species studied belong to the subfamilies *Pamphaginae* and *Thrinchinae*. All species have karyotypes consisting of 19 and 20 acrocentric chromosomes and X0/XX sex chromosome system in males and females, respectively (2n♂=19, NF=19; 2n♀=20, NF=20). Despite the karyotype conservatism, we revealed differences in the location and size of C-heterochromatin blocks and ribosomal DNA clusters. A comparative analysis of these differences shows that karyotype divergences in this group is connected not to structural chromosome rearrangements, but to the evolution of repetitive DNA.

## Introduction

The family Pamphagidae Burmeister, 1840 is a comparatively small group in Acridoidea grasshoppers, which includes 96 genera and 561 species and subspecies (Ünal 2016). The species of this family inhabit the desert, semidesert, and mountainous landscapes of the Palaearctic and Afrotropical Regions (Uvarov 1966, Massa 2013, Ünal 2016). More than 90 species were described in North Africa, most of which occur in Morocco (Massa 2013, Ünal 2016). To date, the Pamphagidae remain one of the least cytogenetically studied groups among the grasshoppers. The early studies of karyotypes of some species from North and South Africa, South-West Europe and East Asia revealed the exceptional karyotype conservatism of this family (Chen 1937, White 1973, Hewitt 1979, Camacho et al. 1981, Santos et al. 1983, Cabrero et al. 1985, Fossey 1985, Fu Peng 1989, Mansueto and Vitturi 1989, Vitturi et al. 1993, Warchałowska-Śliwa et al. 1994). In these studies the diploid sets of chromosomes of Pamphagidae species consisted of 19 (♂) and 20 (♀) acrocentric chromosomes with X0♂/XX♀ sex determination mechanism. It allowed drawing a conclusion about the exceptional karyotype conservatism of this family. Further karyotyping of some Pamphagidae species from Central Asia, Bulgaria and Western and Central Anatolia, however, led to a revision of the notion of a uniform karyotype structure within the family. The vast majority of Pamphagidae species from these regions have a karyotype consisting of 16 acrocentric autosomes and a neo-XY sex chromosome system (2n♂♀=18, neo-XX♀/neo-XY♂) (Bugrov 1996, Bugrov and Warchałowska-Śliwa 1997, Bugrov and Grozeva 1998, Bugrov et al. 2016, Jetybayev et al. 2017). These sex chromosomes arose due to the centric fusion of the original X chromosome with an autosome. In addition, there are karyotypes in which several pairs of chromosomes (*Melanotmethisfuscipennis* (Redtenbacher, 1889)) (Bugrov and Warchałowska-Śliwa 1997) or even all of them (*Eremopezafestiva* (Saussure, 1884)) have short second arms (Bugrov et al. 2016). These indicate that not all Pamphagidae have a conserved chromosomal set, making this group a good model for understanding the karyotype evolution.

The majority of Pamphagidae species that possess a derived karyotype are distributed in Western and Central Asia, which led to assumption that the evolutionary events resulted in karyotypic changes occurred most likely within these territories (Bugrov 1996, Bugrov and Warchałowska-Śliwa 1997, Bugrov and Grozeva 1998, Bugrov et al. 2016, Jetybayev et al. 2017). Unfortunately, scarce cytogenetic data on Pamphagidae from other centers of biodiversity of this family does not allow us to confirm whether such a karyotype derived is characteristic only of the indicated geographical region. For this reason, the study of the species of this family from other areas is indispensable.

In this work, we provide new data on the comparative cytogenetic analysis of some Pamphagidae species from Morocco. To obtain additional information on linear differentiation of chromosomes, we used C-banding and fluorescence *in situ* hybridization (FISH) with (TTAGG)_n_ telomeric and ribosomal DNA probes. The data on the distribution of the C-bands and the clusters of ribosomal DNA and telomeric repeats is available for various insect species (Sahara et al. 1999, Cabrero and Camacho 2008, Grozeva et al. 2011, Jetybayev et al. 2012, Bugrov et al. 2016, Kuznetsova et al. 2017), which provides the base for comparative cytogenetic studies.

## Material and methods

### Material collection

Ten species of Pamphagidae from the High and Middle Atlas in Morocco were collected during May-June 2013. Nine species belong to the subfamily *Pamphaginae* and one to the subfamily *Thrinchinae*. Table 1 describes the material analyzed and the collection localities, as well as the number of individuals examined.

**Table 1.** List of species, collection places and number of specimens of the studied Pamphagidae species.

**Taxa**	**Species**	**Location**	**Number of specimens**
Pamphaginae Pamphagini	*Paracinipealticola* (Werner, 1932)	Morocco (pass N of Taroudant) 30°51.53'N, 8° 22.66'W	2
	*Paracinipecrassicornis* (Bolívar, 1907)	Morocco (Oum Rbia valley) 32°45.40'N, 7°58.33'W	1
	*Paracinipedolichocera* (Bolívar, 1907)	Morocco (El Kebab) 32°45.37'N, 5°38.72'W	2
	*Paracinipetheryi* (Werner, 1931)	Morocco (pass in Antiatlas to Tafraout) 29°49.87'N, 9°2.25'W	2
	*Pseudoglauiatarudantica* (Bolívar, 1914)	Morocco (Antiatlas – pass to Tafraout) 29°49.87'N, 9°2.25'W	1
	*Acinipehespericalepineyi* Chopard, 1943	Morocco (Achahaoud towards road Marakesh-Ouarzazad) 31°15.67'N, 7°23.32'W	1
Pamphaginae Euryparyphini	*Euryparyphesrungsi* Massa, 2013	Morocco (Col du Zad N of Midelt) 33°2.12'N, 5°4.32'W	2
	*Eunapiodesgranosus* (Stål, 1876)	Morocco (NW Ouaourioud) 32°20.41'N 5°43.18'W	2
	*Paraeumigusparvulus* (Bolívar, 1907)	Morocco (pass N of Taroudant) 30°51.98'N, 8°21.48'W	3
Thrinchinae Thrinchini	*Tmethiscisti* (Fabricius, 1787)	Morocco(near Beni Ayadet) 33°41.25'N, 3°40.82'W	2

### Methods

#### Fixation, C-banding and Fluorescence *in situ* hybridization (FISH)

The testes were dissected from adult males and placed into 0.9% solution of sodium citrate for 20 min. The testes were fixed in 3:1 ethanol:glacial acetic acid for 15 min. The fixed material was then rinsed and kept in 70% ethanol. Air-dried chromosome preparations were made by squashing testis follicles in 45 % acetic acid and then freezing them in dry ice.

C-banding of the chromosome preparations was performed according to Sumner (1972) with minor modifications. Chromosome preparations were treated with 0.2 N HCl for 15–20 min, rinsed in distilled water, incubated in a saturated solution of Ba(OH)_2_ at 61 °C for 3–5 min, rinsed in tap water, and then incubated in 2×SSC at 61 °C for 60 min. After being rinsed in distilled water, the slides were stained with 2% Giemsa.

Fluorescence *in situ* hybridization (FISH) with (TTAGG)_n_ telomeric and ribosomal DNA probes on meiotic chromosomes was carried out according to the protocol by Pinkel (1986) with modifications (Rubtsov et al. 2000, 2002). In brief, the slides were treated with 0.1 µg/ml solution of RNAse (Sigma-Aldrich, USA) in 2×SSC for one hour, washed three times in 2×SSC and then dehydrated in 70%, 80% and 96% ethanol for two minutes. After dehydration, the slides were treated with 0.04% pepsin solution (activity ≥ 400 U/mg, Sigma-Aldrich, USA) in 0.01M HCl for 8 minutes at 37 °C, and washed in PBS for 5 minutes, in PBS with 0.1M MgCl for 5 minutes, in 0.1% formaldehyde in PBS with 0.1M MgCl for 10 minutes, and then again in PBS for 5 minutes, and lastly dehydrated in the ethanol series as described above. The 10 µl of the hybridisation solution of 30 ng of each DNA probe and 1 µg of sonicated salmon sperm DNA in hybridisation buffer (50% deionized formamide, 10% dextran sulfate, 2×SSC, 0.01% Tween 20) was applied under cover glass and denaturated on the hotplate for 5 minutes at 75 °C and then hybrised in the humid chamber overnight at 37 °C. The three washing steps were carried out in 50% formamide solution in 2XSSC at 45 °C for 5 minutes, three times in 2XSSC at 45 °C for 5 minutes, three times in 0,2XSSC at 45 °C for 5 minutes and three final times in 0,1XSSC at 65 °C for 5 minutes.

Unlabeled rDNA probe was generated by the polymerase chain reaction (PCR) of six fragments of 18S and 28S rRNA genes using specific primers according to Jetybayev et al. (2017) and Buleu et al. (2017). Because 18S rRNA and 28S rRNA genes are parts of a single 45S rRNA gene, they both were used to detect rDNA cluster. The fragments of the genes were labeled in additional PCR cycles with Fluorescein-12-dUTP (Biosan, Novosibirsk, Russia) and mixed together into a single rDNA probe. Telomeric repeats (TTAGG)_n_ were generated by the non-template PCR method with 5'-TAACCTAACCTAACCTAACC-3' and 5'-TTAGGTTAGGTTAGGTTAGG-3' primers. Further labeling with Tamra-dUTP (Biosan, Novosibirsk, Russia) was performed in additional 33 cycles of PCR as described previously (Sahara et al. 1999).

For the description of chromosomes, karyotypes and C-bands, the nomenclature previously proposed for grasshoppers was used (King and John 1980, Santos et al. 1983, Cabrero and Camacho 1986).

Microscopic analysis was performed at the Centre for Microscopy of Biological Objects (Institute of Cytology and Genetics, Novosibirsk, Russia). Chromosomes were studied with an AxioImager M1 (Zeiss, Germany) fluorescence microscope equipped with filter sets #49, #46HE, #43HE, and a ProgRes MF (MetaSystems GmbH, Germany) CCD camera. The ISIS5 software package was used for image capture and analysis.

## Results

### Karyotype

The diploid sets (2n) of chromosomes all the studied species consist of nine pairs of acrocentric autosomes and one unpaired acrocentric X-chromosome in males and two X chromosomes in females (X0/XX sex determination system). The karyotype structure is represented by of four large (L1–L4), three medium (M5–M7) and two small (S8–S9) pairs of autosomes, and the medium sized X chromosome.

### C-banding

The C-banding of the chromosomes in the studied species reveals three different localizations of the C-blocks: pericentromeric, interstitial and telomeric. The pericentromeric C-bands appear in every species analyzed.

### Pericentromeric C-bands

**Figure 1.**
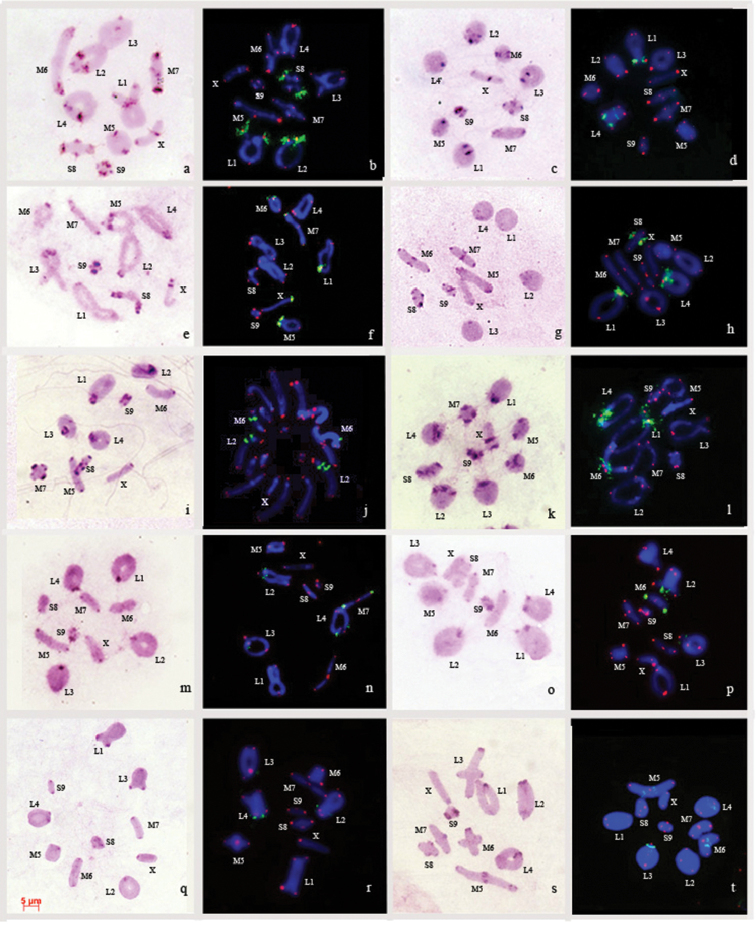
C-banding (**a, c, e, g, i, k, m, o, q, s**) and FISH of rDNA (green) and (TTAGG)_n_ (red) probes (**b, d, f, h, j, l, n, p, r, t**) in meiotic chromosomes of species studied. *Paracinipealticola* (**a, b** diakinesis); *Paracinipecrassicornis* (**c, d** metaphase I); *Paracinipedolichocera* (**e, f** diakinesis); *Paracinipetheryi* (**g, h** diakinesis); *Pseudoglauiatarudantica* (**i** metaphase I **j** spermatogonial metaphase); *Acinipehespericalepineyi* (**k** metaphase I **l** diakinesis); *Euryparyphesrungsi* (**m** metaphase I **n** diakinesis); *Eunapiodesgranosus* (**o, p** metaphase I); *Paraeumigusparvulus* (**q, r** metaphase I); *Tmethiscisti* (**s, t** metaphase I). Scale bar: 5 μm.

The size of the pericentromeric C-bands differs in various chromosomes within the karyotypes of the species studied: most of the chromosomes have small-sized pericentric C-positive blocks, but in some chromosomes medium-sized blocks have been also observed. *Acinipehespericalepineyi* is the only species with medium-sized pericentric C-block in all its chromosomes (Fig. 1k). The medium-sized pericentric C-blocks are also detected in some chromosomes in the following species: *Paracinipealticola* (L4, M5, S8, S9) (Fig. 1a); *P.crassicornis* (L1 – L4, M5, M6) (Fig. 1c); *P.dolichocera* (L3, L4, M6, S8) (Fig. 1e); *P.tarudantica* (L1 – L4, S8) (Fig. 1i); *Euryparyphesrungsi* (L1 – L4) (Fig. 1m); *Eunapiodesgranosus* (L1, L2, L4) (Fig. 1o); *Paraeumigusparvulus* (L1) (Fig. 1q). In *Paracinipetheryi* (Fig. 1g) and *Tmethiscisti* (Fig. 1s), the pericentric C-blocks are of small size in all of the chromosomes.

The X chromosome in all the examined species possesses a small C-heterochromatic block, except for the X chromosome of *P.alticola*, *P.dolichocera* and *A.hespericalepineyi*, which has a medium-sized pericentric C-block (Fig. 1a, e, k).

### Interstitial C-bands

The interstitial C-bands are found in five of the studied species: *P.alticola*, *P.crassicornis*, *P.dolichocera*, *P.theryi* and *A.hespericalepineyi* (Fig. 1a, c, e, g, k). These blocks are of small or medium-sized and are generally localized in the proximal part of the autosomes. In *P.alticola*, medium-sized interstitial C-bands are observed in three large (L1, L2, L4) and one medium (M6) pair of autosomes. In the fourth largest (L4) pair of autosomes, the interstitial C-bands are detected only in one of the homologues. In the middle-sized sixth pair (M6), the interstitial block is dispersed (Fig. 1a). In *P.crassicornis*, a small interstitial block is located in the second largest pair of autosomes (L2) (Fig. 1c). In *P.dolichocera*, the medium interstitial C-bands are identified in a middle (M5) and small (S8) pair of autosomes. The small autosome pair (S8) has a complex of interstitial heterochromatic bands consisting of two or three small blocks. In a large (L3) and medium (M7) autosome pairs, the small-sized interstitial C-bands are detected only in one of the homologues (Fig. 1e). In *P.theryi*, tiny interstitial C-bands are detected in the middle (M5) and large (L2) autosome pairs (Fig. 1g). In *A.hespericalepineyi*, medium-sized interstitial C-bands on two large (L2, L4) and the sixth middle (M6) autosome pairs are found. The third large pair of autosomes (L3) has very small interstitial C-bands (Fig. 1k).

The X chromosome has a medium-sized interstitial C-band in *P.alticola*, *P.crassicornis*, *P.dolichocera* and *A.hespericalepineyi*. The location of interstitial C-bands on the X chromosome differs in these species. In *P.alticola* and *P.crassicornis*, this band is located in the proximal part (Fig. 1a, c), while in *P.dolichocera* and *A.hespericalepineyi* was observed in the distal part of the X chromosome (Fig. 1e, k).

### Telomeric C-bands

Telomeric C-blocks of large, medium and small-sized is revealed in all of the studied species. When telomeric C-bands are present, they are usually located either in medium or small chromosomes.

In *P.alticola*, *P.crassicornis*, *P.dolichocera* and *P.theryi*, telomeric blocks are detected on medium-sized autosomes: two autosome pairs in *P.alticola* (M5, M7) and *P.theryi* (M6, M7) (Fig. 1a, g), and on one chromosome pair in *P.crassicornis* (M6) and *P.dolichocera* (M7) (Fig. 1c, e). The S8 chromosome pair carries telomeric blocks of different sizes in all studied species except for *P.dolichocera*, *E.rungsi*, *E.granosus*, and *P.parvulus* (Fig. 1e, m, o, q). Another autosome pair that has a large telomeric block in all the species studied is the S9 autosome pair. In *P.crassicornis* and *P.tarudantica*, telomeric C-band is located only in one homologue in the S9 autosome pair (Fig. 1c, i). In *T.cisti*, the autosome pairs L1–L4, M5 and S9 have medium-sized telomeric C-bands whereas the M6, M7, and S8 pairs exhibit small-sized telomeric C-blocks (Fig. 1s). Very thin telomeric blocks are detected in M6 and M7 pairs of the *E.granosus* karyotype, and also in L3 and M7 pairs of the *P.parvulus* chromosome set (Fig. 1o, q). The X chromosome has very small-sized telomeric blocks only in *E.rungsi* (Fig. 1m).

### Fluorescence in situ hybridization

#### FISH of telomeric (TTAGG)_n_ DNA probe

FISH experiments with telomeric DNA-probe reveal fluorescent hybridization signals at the ends of all autosome bivalents (Fig. 1b, d, f, h, j, l, n, p, r, t). The telomeric signals are revealed only in the X chromosome of *P.parvulus* in one of its terminal regions (Fig. 1r). The hybridization signals of the telomeric DNA-probe show variation in intensity between chromosomes in the karyotype and among chromosome sets of the studied species (Fig. 1).

#### FISH with the rDNA probe

The cluster of rRNA genes consists of many copies of 45S rRNA gene that are interlaced with non transcribed spacer (Srivastava and Schlessinger 1991). At the same time, 45S rRNA gene contains both 18S and 28S rRNA genes. The FISH experiments of these two DNA probes showed complete colocalization in meiotic chromosomes of all species analysed. The using both DNA probes labeled with same fluorophore as a single rDNA probe allow to show higher intensity of the hybridization signal. Therefore, this combined probe is used to reveal rDNA clusters and to map their distribution and location in further studies.

The clusters of rDNA repeats localize on two (*P.crassicornis*, *P.tarudantica*, *P.parvulus*) (Fig. 1d, j, r), three (*P.alticola*, *P.theryi*, *A.hespericalepineyi*, *E.rungsi* (Fig. 1b, h, l, n) or four (*P.dolichocera*, *E.rungsi*, *T.cisti* (Fig. 1f, n, t) autosome bivalents, and on the X chromosome (*P.dolichocera* (Fig. 1f).

The rDNA repeats are found only in pericentromeric and interstitial regions of chromosomes. The pericentromeric rDNA clusters are detected in karyotypes of all analyzed species except *P.tarudantica* (Fig. 1j). The rDNA is observed in pericentromeric region of the L1 bivalents in all *Paracinipe* species and in *A.hespericalepineyi* (Fig. 1b, d, f, h, l). The L2 bivalent bears rDNA genes at pericentromeric region only in *P.alticola* (Fig. 1b). The pericentromeric region of the L3 bivalents have a single cluster of rRNA genes in *E.granosus* and *T.cisti* (Fig. 1p, t). Hybridization signals are found in L4 bivalents of *P.crassicornis*, *P.theryi*, *A.hespericalepineyi* and *P.parvulus* (Fig. 1d, h, l, r). A single cluster of signals are found in the pericentromeric region of medium autosome bivalents of four species: *P.dolichocera* (M5–M7); *A.hespericalepineyi* (M6) and *E.rungsi* (M7) (Fig. 1f, l, n). In *P.alticola* and *P.theryi*, the probe hybridizes to the pericentromeric region of S8 bivalents (Fig. 1b, h).

Interstitial clusters of rRNA genes are revealed in seven species. The hybridization signals are found in the large autosome bivalents of *P.crassicornis* (L4), *P.tarudantica* (L2), *E.rungsi* (L2, L4), *E.granosus* (L2), *P.parvulus* (L2), and *T.cisti* (L4) (Fig. 1d, j, n, p, r, t). In *P.tarudantica*, the L2 autosome bivalent has two adjacent interstitial rDNA clusters (Fig. 1j). In *T.cisti*, interstitial rDNA clusters are also found in two of the medium bivalents (M5, M6) (Fig. 1t). In the four species *P.tarudantica*, *E.rungsi*, *E.granosus*, *P.parvulus* and *T.cisti*, the rDNA clusters are located in the proximal region of the bivalents (Fig. 1j, n, p, r, t), whereas in *P.crassicornis* (L4) and *P.tarudantica* (M6), the signals were placed in the distal part of the autosome bivalents (Fig. 1d, j). The probe identifies one rDNA cluster only in the X chromosome of *P.dolichocera* at the pericentromeric region (Fig. 1f).

## Discussion

The cytogenetic analysis of the Pamphagidae grasshoppers from Morocco confirmed that the species of Pamphaginae and Thrinchinae subfamilies from the Western Mediterranean region have an exceptionally conservative karyotype consisting of 19 (♂) and 20 (♀) acrocentric chromosomes with a X0♂/XX♀ sex chromosome system. Previously, we described five species of Pamphagidae (*Eunapiodesatlantis* (Chopard, 1943), *Paraeumigusfortius* (Bolivar, 1907), *Euryparyphesflexuosus* Uvarov, 1927, *Acinipetubericollis* Werner, 1932, and *Pseudoglauiaterrea* (Bolivar, 1912)) from the same region and showed that these species had a male and female diploid chromosome number of 19 and 20, respectively with standard type of sex chromosome system (X0♂/XX♀) (Buleu et al. 2015). These results indicate that only the Nocarodeini tribe of Pamphaginae subfamily which prevails in Western Asia, the Caucasus and Transcaucasia, has a 2n=18, XY♂/XX♀ karyotype (Bugrov et al. 2016, Jetybayev et al. 2017).

In general, the collected data on the distribution of C-heterochromatin in chromosomes of the species studied agrees with the results of certain Pamphagidae species from Spain (Camacho et al. 1981, Santos et al. 1983, Cabrero et al. 1985). The difference in size and localization of C-positive blocks in several species allow proposing that the repetitive DNA sequences would be the responsible of the existing diversity of karyotypes in this group, and not the structural rearrangements of chromosomes. The analysis of the C-banding revealed three different chromosomal positions of the C-positive blocks: pericentromeric, interstitial and telomeric. The pericentromeric and telomeric C-heterochromatic blocks were detected in all species analysed, whereas interstitial C-positive blocks were observed only in four species of the genus *Paracinipe* and in *Acinipehespericalepineyi*. In most of the species of the present work, the pericentromeric block was small-sized. However, in some of the species, large, medium or small bivalents had a medium-sized pericentromeric block. In previously species studied from the same region (*E.atlantis*, *P.forties*, *A.tubericollis*, *P.terrea*), we revealed similar sized pericentromeric blocks in large pairs of autosomes (Buleu et al. 2015). Occasionally small- or medium-sized interstitial blocks were placed in the proximal part of medium (M5 in *P.theryi*; M6 in *P.alticola*) and small (S8 in *P.dolichocera*) bivalents. These dispersed interstitial blocks were located in close proximity to the near pericentromeric region. Similar interstitial C-blocks were previously observed on medium and small autosomes pairs in *Pseudoglauiaterrea* (Buleu et al. 2015). It was suggested that findings of such blocks support the hypothesis that the differences in the size of C-blocks may be caused by addition (or loss) of heterochromatin (Camacho et al. 1981).

Besides, the X chromosome of four species, *P.alticola*, P.cf.crassicornis, *P.dolichocera*, and *A.hespericalepineyi*, had an interstitial C-positive block. The interstitial block in the X chromosome of *P.alticola* and P.cf.crassicornis was located in its proximal part, whereas in *P.dolichocera* and *A.hespericalepineyi* it was in the distal part of the X chromosome. It is possible that the relocation of this block was caused by inversions (Jetybayev et al. 2012).

Telomeric C-heterochromatin blocks were detected in all of the studied species in one or two medium or small autosome bivalents. Only in *E.rungsi*, the X chromosome had a very small size telomeric block. The small bivalent (S9) had large or medium-sized telomeric C-blocks in all studied species. The presence of large telomeric C-blocks in these small bivalents have already been observed in Pamphagidae (Camacho et al. 1981, Bugrov and Warchałowska-Śliwa 1997, Bugrov et al. 2016, Jetybayev et al. 2017).

Furthermore, in L4 of *P.alticola*, and in L4 and M7 of *P.dolichocera*, the interstitial C-bands were detected only in one of the homologues of these bivalents (Fig. 1a, e). This observation may indicate the presence of a polymorphism in the populations of these species. However, the study of a small number of specimens of the same species does not allow drawing conclusions about C-band polymorphisms, as found in *Asiotmethisheptapotamicus* (Bugrov et al. 2016).

The FISH analysis with (TTAGG)_n_ probe revealed that this DNA motif is a component of the telomeres in all chromosomes of species herein studied. This motif is widespread through different lineages of insects and other arthropods, and it is considered as the ancestral sequence of telomeres in chromosomes of arthropods (Vítková et al. 2005, Traut et al. 2007). FISH with the telomere DNA probe revealed a variation in the intensity of hybridization signals among chromosomes in the karyotype and among chromosome sets of the species studied. These variations may be associated with the peculiarities of the labeled probe penetration through the cell cytoplasm during the FISH, or with the quantity of telomeric DNA repeats. The presence of interstitial telomeric sequences (ITSs) was not revealed. This fact may indicate the absence of structural rearrangements involving terminal regions in Pamphaginae karyotype evolution. Such rearrangements were previously detected in Acrididae grasshoppers (Jetybayev et al. 2012).

In previous studies, rDNA genes was mapped using plasmid containing complete 45S rDNA (Cabrero and Camacho 2008), or 18S rDNA (Jetybayev et al 2012), or 28S rDNA (Buleu et al. 2017). In current study, we used the 18S and 28S DNA probes and observed complete colocalization of the signals from 18S and 28S rDNA. These results confirm that 18S and 28S rRNA genes are in fact parts of single 45S rRNA cluster.

In this study, rDNA-FISH revealed an interspecific variation in the localization of ribosomal genes. In most of the species, the rDNA cluster were located at the pericentric region in the large bivalents and in the fifth, sixth and seventh medium ones. In *P.alticola* and *P.theryi*, the rDNA clusters were also mapped at the pericentric region of small chromosomes (S8). The ribosomal clusters at interstitial regions of large bivalents (L2, L4) were revealed in *P.crassicornis*, *E.rungsi*, *E.granosus*, *P.parvulus* and *T.cisti*. In *P.tarudantica* and *T.cisti*, interstitial rDNA genes was detected in the medium autosome bivalents M5 and M6. Usually these bivalents have a single interstitial rDNA cluster. However, two interstitial rDNA clusters were located in one large chromosome pair (L2) of *P.tarudantica*. Multiple localization of rDNA clusters in a single chromosome was previously reported for *Pamphagusortolaniae* (Vitturi et al. 2008), *Pseudoglauiaterrea* (Buleu et al. 2015) and some Pamphagidae species from Armenia (Bugrov et al. 2016) and Turkey (Jetybayev et al. 2017). It should be emphasized, that the multiple localization of rDNA clusters in a single chromosome among Acridoid grasshoppers has been detected so far in species of the Pamphagidae family exclusively (Jetybayev et al. 2017, Buleu et al. 2017). In the cytogenetically well-studied family Acrididae, the distribution of rDNA clusters was limited mainly to the one or two pair of chromosomes per karyotype (Cabrero and Camacho 2008, Jetybayev et al. 2012, Palacios-Gimenez et al. 2013). The multiple rDNA clusters in Pamphagidae may support the hypothesis of mutual translocations of two pairs of autosomes in the 19-chromosome karyotype of Pamphagidae from the basal 23-chromosome karyotype of Acridoidea (White 1973). Probably, two ancestral pair of chromosomes carried rDNA clusters and they formed a chromosome pair with two clusters of rDNA after the fusion. This hypothesis is based on that rDNA clusters usually tend to localize in pericentric or proximal regions (Cabrero and Camacho 2008, Loreto et al. 2008, Cabral-de-Mello et al. 2011, Jetybayev, 2012, Palacios-Gimenez et al. 2013). Therefore, if the initial fusion were centric, two rDNA clusters would lie very close to each other or even fuse into one cluster. In the case under consideration, however, all the observed double rDNA clusters were clearly distinct, thus the fusion would be a tandem one and not centric.

A pericentromeric rDNA cluster in the X chromosome were only found in one species (*P.dolichocera*). Conversely, it is worth noting that the presence of ribosomal genes in the X chromosome was reported for many grasshoppers (Cabrero and Camacho 2008, Cabrero et al. 2009, Veltsos et al. 2009). In Pamphagidae species with an X0/XX sex system, the rDNA loci was found in the X chromosome of only two species, namely *Asiotmethismuricatus* (Pallas, 1771) and *A.tauricus* (Tarbinsky, 1930) (unpublished data). Among the Pamphagidae species that have neoXY/neoXX sex chromosome system, the neo-X often carries rDNA cluster and they are usually located at interstitial region (Jetybayev et al. 2017). This observation may indicate evolutionary changes that have occurred in the X chromosomes. Possible mechanisms explaining changes in rDNA cluster location could be paracentric inversion, or insertion of DNA fragments containing rDNA into the chromosome, with subsequent rDNA amplification and elimination of the old rDNA cluster, or transposition of the NOR region (i.e. interchromosomal mobility of NOR regions) (Arnheim et al. 1980, Schubert and Wobus 1985, Dubcovsky et al. 1995, Roy et al. 2005, Cabrero and Camacho 2008).

In conclusion, in spite of the karyotypic conservatism of the Pamphagidae species studied, cytogenetic differences in the location of chromosome markers (C-heterochromatin blocks, telomere sequences and ribosomal genes) were found in both closely related species of one genus and between different genera. The differences in localization of these cytogenetic markers in closely related species appear to be associated with chromosomal rearrangements known to play a fundamental role in speciation (White 1968). Since many Pamphagids have a standard set of chromosomes, these changes need to be taken into account to explain the speciation processes within and between genera.
